# Evolving Cystic Fibrosis Care: Lung Immunology and Emerging Health Challenges in the Era of CFTR Modulators

**DOI:** 10.3390/biom15101460

**Published:** 2025-10-16

**Authors:** Giuseppe Fabio Parisi, Maria Papale, Giulia Pecora, Santiago Presti, Monica Tosto, Enza Mulé, Vittorio Ornato, Donatella Aloisio, Salvatore Leonardi

**Affiliations:** Pediatric Respiratory Unit, Department of Clinical and Experimental Medicine, San Marco Hospital, University of Catania, 95121 Catania, Italy; m.papale@policlinico.unict.it (M.P.); giupec87@hotmail.it (G.P.); santiago.presti@policlinico.unict.it (S.P.); monitosto@gmail.com (M.T.); enzamule73@gmail.com (E.M.); vittorioornato@icloud.com (V.O.); donatellaaloisio@gmail.com (D.A.); leonardi@unict.it (S.L.)

**Keywords:** cystic fibrosis, CFTR modulators, lung immunology, microbiome, systemic complications, cancer risk, CF-related diabetes, long-term management, personalized medicine, integrated care

## Abstract

The introduction of CFTR modulators has dramatically shifted the clinical management of cystic fibrosis (CF) from a life-limiting pediatric condition to a chronic disease with broader health implications. This review explores the impact of these advancements on lung immunology and the emerging spectrum of health challenges. While these modulators have reduced traditional pulmonary complications by mitigating inflammation and infection, they also introduce new considerations for long-term health management. As patients experience longer lives, issues such as the increased risk of certain cancers and other systemic complications like CF-related diabetes and liver disease are gaining attention. Understanding the interplay between CFTR modulators, immune response, and the development of these conditions is essential for optimizing patient outcomes. This review highlights the importance of integrated care strategies that address both the respiratory improvements and emerging health risks associated with longer life expectancy in CF patients. By fostering a comprehensive approach, we aim to enhance the overall quality of life and address the complex needs of individuals navigating CF in the modern therapeutic landscape.

## 1. Introduction

**Figure 1:**
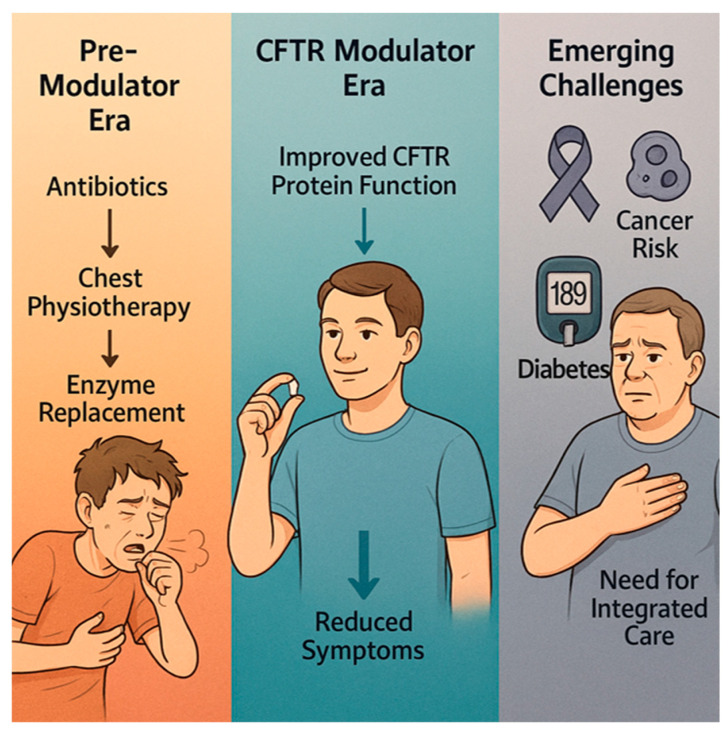
The evolving landscape of cystic fibrosis (CF) care across three eras: the Pre-Modulator Era, the CFTR Modulator Era, and the era of Emerging Challenges.

The landscape of cystic fibrosis (CF) has undergone a profound transformation following the advent of Cystic Fibrosis Transmembrane Regulator (CFTR) modulator therapies, which target the underlying genetic defect rather than solely managing symptoms. These therapies—such as ivacaftor, lumacaftor, tezacaftor, and elexacaftor—have demonstrated remarkable efficacy in restoring partial CFTR function, leading to significant improvements in pulmonary outcomes and extending median life expectancy well into the fourth or fifth decade [1,2]. This shift has profoundly altered the clinical trajectory of CF, shifting the focus from life-limiting respiratory failure to managing a chronic multisystem disease with complex long-term implications [3,4,5]. However, the extended lifespan of CF patients has revealed new health challenges. Notably, recent evidence suggests a potential association between CFTR modulation and alterations in immune response and systemic inflammation, which may influence the development of comorbidities that are traditionally less emphasized in CF, such as cancer and metabolic diseases [6,7,8]. For instance, data indicate shifts in the epidemiology of malignancies, with increased incidences of certain gastrointestinal and hematological cancers, possibly related to chronic inflammation, immune surveillance changes, or long-term effects of modulator therapy itself [9,10]. Furthermore, understanding how CFTR modulators modulate immune responses—both in the lungs and systemically—is vital, as these changes may impact susceptibility to infection, inflammatory status, and even tumorigenesis. Simultaneously, evolving management challenges include optimizing treatment strategies for extrapulmonary manifestations and understanding long-term safety profiles [11,12] [Figure 1].

This review aims to synthesize current insights into how CFTR modulators influence lung immunology within the context of a longer-living CF population, addressing emerging systemic health concerns and highlighting future directions for personalized, multidisciplinary management.

## 2. Materials and Methods

This review is based on a comprehensive qualitative analysis of published literature pertinent to the impact of CFTR modulators on lung immunology and systemic health in CF. The literature search was performed using scientific databases including PubMed, Scopus, and Google Scholar, covering publications from 2010 to 2024. Keywords used included “cystic fibrosis,” “CFTR modulators,” “lung immunology,” “systemic complications,” and “long-term effects.”

Selection criteria included peer-reviewed original articles, reviews, and meta-analyses that addressed the immunological alterations induced by CFTR modulators, and studies exploring systemic health issues such as cancer risk, metabolic effects, and other extrapulmonary complications. Articles were filtered based on relevance, methodological quality, and date of publication, prioritizing recent evidence from clinical trials and experimental studies. All data, figures, and key references cited in this review are available within the publication’s reference list.

No new experimental data or primary datasets were generated or deposited for this review. In line with recent guidelines, this manuscript discloses that artificial intelligence tools (OPEN AI—GPT5) were used solely for language editing and formatting; no AI-driven analysis, data generation, or study design support was employed.

## 3. Results

### 3.1. Effects of CFTR Modulators on Pulmonary Immune Response

#### 3.1.1. Modulation of Inflammatory Mediators

Recent evidence indicates that CFTR modulators—particularly highly effective combinations such as elexacaftor/tezacaftor/ivacaftor—produce measurable reductions in airway inflammation in people with cystic fibrosis. At the biochemical level, treatment is commonly associated with decreases in canonical pro-inflammatory cytokines and chemokines (notably IL-8 and IL-6), which are central drivers of neutrophil recruitment and activation [13]. Parallel declines have been observed in neutrophil-derived effectors and markers of oxidative stress, including neutrophil elastase, myeloperoxidase and markers of neutrophil extracellular traps (NETs). Changes have also been reported in matrix-remodeling enzymes (e.g., certain MMPs) and in systemic acute-phase reactants such as CRP, suggesting that modulation of airway inflammation may be reflected, to varying degrees, systemically [12,14].

Mechanistically, several processes are likely to contribute to these anti-inflammatory effects. Partial restoration of CFTR function improves airway surface liquid hydration and mucociliary clearance, reducing the retention of mucus and the microbial and particulate stimuli that chronically activate epithelial and immune cells. Corrected epithelial ion transport and reduced cellular stress signaling (for example, diminished NF-κB activation) decrease epithelial production of neutrophil chemoattractants and alarmins. In parallel, modulator therapy appears to favorably alter innate immune cell function — for instance, promoting more effective phagocytosis by macrophages and reducing excessive neutrophil degranulation—which together reduce the feed-forward cycle of inflammation and tissue injury [15,16]. Temporal patterns of biomarker response are heterogeneous. Some inflammatory mediators fall rapidly within weeks of therapy initiation and correlate with early clinical benefits (improvements in FEV1, weight gain, and fewer pulmonary exacerbations). Other markers decline more slowly or remain elevated for months to years in many patients, reflecting persistent structural lung damage, entrenched microbial colonization, or individual host factors (baseline disease severity, prior treatment history, and genotype). This incomplete normalization suggests that while CFTR correction substantially mitigates drivers of inflammation, it does not uniformly erase pre-existing pathological changes. The clinical implications are manifold. The observed biochemical shifts support the concept that CFTR modulators address a root cause of inflammation and may reduce cumulative inflammatory burden over time, potentially slowing structural decline. At the same time, residual inflammation in a subset of patients argues for adjunctive strategies—such as targeted anti-inflammatory agents or therapies addressing biofilm and persistent pathogens—rather than assuming modulators alone are universally sufficient. From a research and clinical-monitoring perspective, there is a clear need for standardized longitudinal biomarker panels (airway and systemic) to track treatment response, identify patients with persistent inflammation, and guide individualized escalation or de-escalation of anti-inflammatory therapies [17,18]. This modulation results in a marked reduction in neutrophil chemoattractants, which plays a crucial role in minimizing excessive neutrophilic infiltration—a central contributor to tissue damage in CF lungs. Concurrently, reductions in proteolytic enzymes like neutrophil elastase have been observed, which are directly involved in airway remodeling, destruction of elastic tissue, and mucus hypersecretion. These biochemical changes collectively suggest that CFTR modulators do more than just restore ion transport; they intervene in the inflammatory cascade that sustains and worsens lung damage [19,20,21].

Despite these promising findings, it remains uncertain whether these anti-inflammatory effects are sustained long-term at a level sufficient to fully resolve airway inflammation. Some evidence indicates residual inflammatory activity persists even in patients on continuous therapy, implying that CFTR correction alone may not entirely eliminate the chronic inflammatory state. Therefore, understanding the extent and durability of these anti-inflammatory effects is vital for optimizing patient management and developing complementary therapies [22,23].

#### 3.1.2. Changes in Immune Cell Profiles

The effects of CFTR modulators on immune cell populations within the CF lung environment are intricate and remain an active area of research. A key observation in recent studies is a reduction in neutrophil infiltration and activity following therapy. Neutrophils, which are the dominant inflammatory cells in CF airways, are known to produce proteases, reactive oxygen species, and inflammatory cytokines that contribute to tissue damage. After treatment with CFTR modulators, markers of neutrophil activation—such as myeloperoxidase and elastase—are often decreased, coinciding with improvements in clinical measures such as lung function and symptom control [20,24].

In addition to changes in neutrophil biology, macrophages—key coordinators of innate defense and tissue homeostasis in the lung—also appear to undergo notable functional reprogramming following CFTR modulator therapy. Multiple lines of evidence indicate that alveolar and airway macrophages show enhanced phagocytic capacity after modulation, with improved uptake and killing of bacteria and more efficient clearance of apoptotic cells and debris (efferocytosis). This improvement may reflect both a more favorable airway microenvironment (improved hydration, less viscous mucus, reduced inflammatory mediators) and cell-intrinsic effects of partially restored CFTR function on macrophage physiology. Phenotypically, treated macrophages often shift away from a strongly pro-inflammatory profile toward a state more consistent with resolution and repair. This can include reduced production of pro-inflammatory cytokines and chemokines, decreased propensity for prolonged inflammasome activation, and increased expression of markers associated with tissue remodeling and anti-inflammatory signaling. Such a shift helps break the self-sustaining cycle in CF airways where persistent macrophage activation perpetuates neutrophil recruitment and tissue injury. At the mechanistic level, several processes may underlie these functional changes. Improved epithelial function and reduced epithelial alarmin release (e.g., diminished DAMPs) likely lower persistent macrophage stimulation. CFTR correction in macrophages themselves may also alter ion fluxes, endosomal pH, and phagolysosomal function, thereby improving intracellular killing of bacteria and reducing chronic activation [25]. Metabolic reprogramming of macrophages—moving from a glycolytic, pro-inflammatory state toward oxidative metabolism associated with resolution—has been proposed as another contributing factor. The consequences of macrophage reprogramming are clinically relevant. Enhanced phagocytosis and reduced inflammatory signaling can decrease bacterial load, limit protease release, and lower collateral tissue damage, contributing to improved lung function and fewer exacerbations. Improved efferocytosis may also reduce the accumulation of necrotic material that fuels biofilm persistence and inflammation. However, the degree of improvement is heterogeneous; in patients with advanced structural damage or entrenched biofilms, macrophage recovery may be partial, and dysfunctional populations can persist. Important uncertainties remain. It is not yet clear how durable macrophage phenotype changes are over years of therapy, whether all macrophage subsets respond similarly (alveolar vs. interstitial vs. recruited monocyte-derived macrophages), or how these shifts interact with adaptive immunity and long-term infection dynamics. Methodological differences (source of cells, in vitro vs. ex vivo assays, patient age and disease stage) complicate comparisons across studies [26,27].

However, some evidence suggests that although neutrophil numbers decrease, residual neutrophils may still persist and participate in ongoing inflammatory pathways. These remaining cells might be more resistant to resolution signals or could be contributing to low-level, chronic inflammation. This residual activity indicates that CFTR correction does not fully restore a normal immune environment, and some inflammatory processes may continue despite therapy [24,28].

Furthermore, alterations are observed in adaptive immune responses, notably with changes in T-cell populations. An increase in T-helper 1 (Th1) profile and a balanced Th1/Th2 response indicate modulation of adaptive immunity, which could influence infection control and inflammatory resolution. The shift toward a more regulated immune response may reduce the likelihood of hyperinflammation but might also alter host defense mechanisms against pathogens [29].

These immune profiling efforts, often employing techniques like flow cytometry and transcriptomics, reveal the complex and nuanced effects of CFTR modulators. They underscore the potential for these therapies to restore immune balance but also highlight the need for further research to fully understand how immune cells adapt to and are modulated by long-term treatment [30].

#### 3.1.3. Impact on Infection Susceptibility

One of the most complex and evolving aspects of corticosteroid therapy in CF relates to how CFTR modulators influence the risk and nature of respiratory infections. Historically, CF patients suffer from chronic colonization or infections by pathogens such as Pseudomonas aeruginosa, Staphylococcus aureus, and increasingly, other multidrug-resistant organisms [31]. The primary goal of current therapies has been to reduce mucus viscosity and improve mucociliary clearance, which ideally should lead to decreased bacterial load and infection severity. However, emerging data indicate that the relationship between CFTR modulation and infection dynamics is not straightforward [32,33]. While some patients show a marked reduction in pathogen load and fewer exacerbations, others exhibit a transient or persistent increase in bacterial colonization after initiating CFTR therapy. In some cases, certain bacteria—especially biofilm-forming organisms—appear to persist or even proliferate, possibly due to changes in the immune landscape or microbiome composition induced by treatment [34].

Mechanistically, the impact of CFTR modulators on the immune system’s ability to clear bacteria is complex and multifaceted. While these therapies effectively reduce neutrophil-driven tissue damage by decreasing neutrophil recruitment and activation, they may inadvertently influence other aspects of host defense. For instance, neutrophils, although primary mediators of inflammation, also play a crucial role in phagocytosing and eliminating bacteria, particularly in the context of biofilms and persistent colonizations characteristic of CF lungs [35]. With modulator therapy, improvements in airway function and reductions in bacterial burden may lead to decreased neutrophil activity, which could secondarily affect bacterial clearance. However, the clinical significance of this potential effect is complex. In patients with established chronic colonization or bacteria residing in biofilms, which are inherently more resistant to immune attack and antibiotics, clearance is often limited regardless of modulator-induced changes in neutrophil activity. In these cases, neutrophil activity may remain elevated due to persistent immune stimulation [23,35]. Consequently, a reduction in neutrophil degranulation and oxidative burst activity might provide a double-edged sword—reducing tissue damage on one hand but potentially allowing certain pathogens to persist or even expand, due to diminished immune pressure. Furthermore, changes in airway surface liquid composition—such as improved hydration and mucociliary clearance—can alter local immune signaling and microbial behavior. For example, a more normalized mucus environment might suppress bacterial virulence gene expression or biofilm formation, but it could also unintentionally create conditions favoring the persistence of some bacteria or the emergence of more virulent strains. These shifts in host–microbe interactions are influenced by multiple factors, including epithelial cell signaling, local cytokine milieu, and microbial adaptation mechanisms. Overall, while CFTR modulators promote a less inflamed and more tissue-preserving environment, they also introduce new challenges in balancing immune responses to optimize pathogen clearance without exacerbating tissue injury. This underscores the need for ongoing research to better understand how these therapies influence both host defense and microbial ecology, to develop adjunctive strategies that preserve effective immunity while minimizing inflammation and tissue damage [36,37]. Researchers are actively investigating various biomarkers to better understand and monitor lung disease progression in CF. These biomarkers can provide valuable insights into the underlying inflammatory processes and help guide treatment strategies. The role of YKL-40 as a potential indicator of lung disease severity in CF is supported by studies such as that of Leonardi et al., who observed a correlation between elevated YKL-40 levels and increased frequency of pulmonary exacerbations [28]. These markers, when considered together, can provide a more comprehensive picture of the inflammatory landscape in the CF lung [38].

Additionally, the airway microbiome, which is highly diverse and dynamic, appears to undergo changes in composition following treatment. Microbial diversity may increase or decrease depending on individual responses, affecting the ecosystem’s resilience and susceptibility to further colonization. These alterations could influence the evolution of bacterial populations, potentially leading to the selection of more resistant strains or alternative pathogenic species [39,40].

Understanding the complex interaction between CFTR modulation, immune responses, and microbiota dynamics is crucial for developing adjunctive strategies aimed at infection control. This includes reevaluating antimicrobial stewardship, considering personalized microbiome-targeted therapies, and monitoring pathogen profiles longitudinally in patients undergoing CFTR therapy. Ultimately, a comprehensive approach that integrates immune modulation, microbiome management, and infection prevention strategies is necessary to fully optimize clinical outcomes in the era of highly effective CFTR modulators [32].

### 3.2. Systemic Effects and Emerging Complications

As CF patients now experience longer lifespans due to advances in CFTR modulator therapies and comprehensive care, a new spectrum of systemic health issues is emerging, requiring increased clinical attention. These complications extend beyond the airways and digestive system, impacting multiple organ systems and influencing overall prognosis. It is important to note that the increasing age of the CF population also contributes to the emergence of these conditions, as many comorbidities, such as cancer, CF-related diabetes, and liver disease, naturally increase with age. Attributing the observed increase in incidence solely to CFTR modulators without considering the effects of aging would be an overinterpretation of the available data [41].

#### 3.2.1. Increased Cancer Risk

The potential rise in cancer incidence among cystic fibrosis patients has become a focal point of recent research, as patients live longer and accumulate various systemic exposures over time. However, it is crucial to interpret these trends with caution. The incidence of many cancers increases with age, and without age-matched epidemiological comparisons to the general population, it is difficult to determine the extent to which the observed rise is attributable to CFTR modulators versus the natural aging process. Future research is needed to conduct such comparisons and to elucidate the specific mechanisms by which CFTR modulators may influence cancer risk in the long term. Historically, cancer prevalence in CF was considered low due to the relatively short lifespan; however, emerging epidemiological data indicate a gradual increase in the occurrence of certain malignancies, notably colorectal cancer, lymphomas, and gastrointestinal tumors. This trend appears to be exacerbated by prolonged exposure to chronic inflammation, immune dysregulation, and environmental factors inherent to CF management [8].

Chronic inflammation plays a dual role, promoting tissue damage while also creating a microenvironment conducive to tumor initiation and progression. In CF, persistent airway and gastrointestinal inflammation, compounded by immune modulation from CFTR therapies, may contribute to DNA damage and oncogenic transformation. Moreover, alterations in the microbiome, with shifts in bacterial populations and decreased microbial diversity, might influence carcinogenic processes by affecting local immune responses and metabolic pathways [8,42,43,44].

Long-term antibiotic use, corticosteroids, and other therapies also warrant consideration, as they can induce DNA damage or select for resistant microbial strains with potential oncogenic implications. For example, some studies have noted an increased risk of colorectal carcinoma, particularly in patients over 40, emphasizing the importance of early screening. The recognition of this heightened risk necessitates the implementation of tailored screening protocols—such as colonoscopy starting at an earlier age—and preventive strategies to mitigate modifiable risk factors [45,46,47].

Furthermore, understanding the biological mechanisms underlying this increased cancer risk remains a priority. Investigations into genetic susceptibilities, immune response alterations, and microbiome–host interactions will be instrumental in developing targeted interventions to reduce cancer morbidity and improve long-term outcomes in the CF population.

#### 3.2.2. Other Systemic Manifestations

Beyond cancer, prolonged survival has revealed a range of other systemic issues previously under-recognized in CF. These systemic effects, which we have already touched upon, also include the complex interplay between CFTR modulator therapy and other common CF manifestations, such as digestive dysfunction and CF-related diabetes (CFRD). For example, while CFRD remains a common comorbidity, partially driven by pancreatic damage and now potentially exacerbated by metabolic effects of newer therapies [6], emerging evidence suggests that CFTR modulators can have varied and interconnected effects, with some studies indicating potential for improved insulin secretion influencing glucose metabolism, while others suggest a possible unmasking of underlying glucose intolerance, further impacting nutritional status and overall metabolic health, necessitating careful monitoring [48]. Similarly, while not fully resolved, some improvements in digestive function, such as increased fat absorption affecting weight gain and overall nutrition, have been observed with modulator therapy in certain patients, though many still require enzyme replacement, influencing their ability to maintain a healthy BMI and absorb essential nutrients [49]. Liver disease, including cirrhosis, continues to be a significant cause of morbidity, with some studies indicating possible links between immune modulation and hepatic pathology [50]. Osteoporosis and bone fractures are also increasingly reported, likely related to malabsorption, chronic inflammation, and physical inactivity [51]. Cardiovascular health, renal function, and neurocognitive outcomes are additional areas warranting ongoing evaluation [52].

#### 3.2.3. Long-Term Safety and Unknowns

While CFTR modulators have brought remarkable benefits, their long-term safety profile remains incompletely understood. Potential adverse effects, such as unforeseen impacts on immune surveillance, carcinogenesis, and organ function, are the subject of current research. Moreover, the interplay between these therapies and the evolving immune landscape, microbiota, and metabolic systems could introduce new risks or modify existing ones. Continuous longitudinal monitoring, registries, and prospective studies are essential to identify and mitigate these issues, ensuring that the extension of survival translates into sustained quality of life [53,54].

### 3.3. Microbiome and Lung Environment

#### 3.3.1. Microbiome Composition Changes

Recent research has increasingly demonstrated that the lung microbiome in CF patients is dynamic and responsive to various treatments, especially with the widespread adoption of CFTR modulators. Historically, CF lungs have been characterized by relatively low microbial diversity, with dominance by pathogenic bacteria such as Pseudomonas aeruginosa, Staphylococcus aureus, Burkholderia cepacia complex, and other opportunistic organisms. This pathogenic dominance often correlates with increased inflammation, tissue damage, and frequent exacerbations [39,40,55].

With the advent of highly effective CFTR modulators—such as elexacaftor/tezacaftor/ivacaftor—there has been increasing evidence suggesting a shift in the airway microbial ecosystem. Several studies indicate that, following therapy initiation, there is a notable increase in microbial diversity within the lower airways. This change is significant because a more diverse microbiome is generally associated with a healthier lung environment in both CF and non-CF populations [37,56,57].

The observed increase in diversity likely results from improved mucus clearance, which reduces the accumulation of thick, viscous mucus that provides a breeding ground for pathogenic bacteria. Better mucociliary function, combined with immune modulation, may create a less hostile environment for benign or commensal bacteria, thus allowing a broader array of microorganisms to coexist within the airway ecosystem [39,55,56].

Furthermore, some studies report a decline in the relative abundance of Pseudomonas aeruginosa and other dominant pathogens. At the same time, there appears to be an increase in bacteria typically associated with a healthy respiratory tract, such as species from the genera Prevotella, Veillonella, and Rothia. This shift towards a more balanced microbiota has been linked with reductions in airway inflammation markers, such as neutrophil counts and pro-inflammatory cytokines, ultimately correlating with clinical improvements [58,59].

However, the extent and durability of these microbiome changes are still under investigation. While some patients maintain a more diverse and less pathogenic microbial community over time, others experience fluctuations influenced by factors such as antibiotic usage, exacerbations, and disease progression. Persistent colonization by some pathogens, especially biofilm-forming bacteria, can still pose challenges despite the overall trend towards greater diversity [60].

Lastly, understanding these microbiome dynamics has important therapeutic implications. It opens the possibility of microbiota-targeted interventions—such as probiotics, prebiotics, or microbiome modulation therapies—to sustain beneficial microbial communities and further reduce inflammation and infection risk. Overall, while promising, long-term studies are needed to elucidate the stability of microbiome shifts and their precise impact on disease trajectory in the era of highly effective CFTR therapies [61].

#### 3.3.2. Correlation with Inflammatory Status

The interplay between the lung microbiome and inflammation in cystic fibrosis is complex and significantly influences disease progression. A diverse microbial community, rich in benign and commensal bacteria such as Prevotella and Veillonella, has been associated with a subdued inflammatory response, likely due to their ability to inhibit pathogenic colonization and modulate immune activity. Conversely, dominance by specific pathogens like Pseudomonas aeruginosa and Staphylococcus aureus often correlates with increased cytokine levels—such as IL-8 and TNF-α—and elevated neutrophil activity, leading to tissue damage and faster lung deterioration [62,63].

In patients treated with CFTR modulators, a notable shift toward a more balanced microbiota corresponds with reductions in pro-inflammatory mediators and neutrophil-derived enzymes, indicating an environment that favors tissue healing. Nevertheless, residual pathogenic bacteria persist in some cases, and fluctuations in microbial composition over time are common, influenced by antibiotic exposure, environmental factors, and individual variability [57,64].

Understanding how microbiome alterations impact inflammation opens avenues for targeted therapies, such as microbiota modulation, to further enhance immune regulation and reduce exacerbations. These strategies could ultimately help sustain lung health by maintaining a microbiome structure that supports immune balance and limits chronic inflammation.

As summarized in Table 1, Section 3 highlighted how CFTR modulators influenced pulmonary inflammation and microbiota, which are intricately linked to systemic health outcomes.

## 4. Discussion

The landscape of CF treatment and understanding has fundamentally changed in recent years, largely thanks to the advent of highly effective CFTR modulators. These therapies have revolutionized the prognosis for many patients, significantly improving lung function, reducing exacerbations, and extending lifespan. However, as survival extends into older age groups, a new and more complex picture has emerged, revealing systemic effects and complications that were previously underappreciated [53].

At the pulmonary level, evidence indicates that CFTR modulators influence the inflammatory environment profoundly. The reductions in pro-inflammatory cytokines and neutrophil activity, along with the normalization of immune cell profiles like macrophages, suggest that correcting the fundamental defect impacts immune responses at multiple levels. Nonetheless, residual inflammation persists in many patients, hinting at the limits of current therapies to fully restore immune balance. This residual activity raises important questions about whether additional anti-inflammatory strategies might be required to prevent ongoing tissue damage and slow disease progression [42,57]. Moreover, the impact of these therapies on the lung microbiome has been promising; increased microbial diversity and a shift away from pathogenic dominance are associated with decreased inflammation and better functional status. Still, microbiota remains a highly dynamic ecosystem, variable across individuals and fluctuating over time, and understanding how to maintain beneficial microbial states long-term remains an open challenge [56,62,65].

Beyond the lungs, systemic effects are gaining prominence as survival improves. The increase in cancer incidence, especially gastrointestinal and hematological malignancies, reflects underlying chronic inflammation, immune dysregulation, and perhaps the influence of long-term therapies and microbiome alterations. This highlights the importance of developing targeted screening and preventive measures, as well as deepening our understanding of the biological mechanisms linking systemic inflammation and carcinogenesis. Concurrently, other systemic manifestations such as cystic fibrosis-related diabetes, liver disease, osteoporosis, and cardiovascular risks are becoming more evident. They underscore the necessity for a truly multidisciplinary approach that anticipates and manages multisystem complications, rather than focusing solely on respiratory health [66].

The overarching challenge moving forward is to integrate these insights into personalized, holistic care strategies. Long-term longitudinal studies will be essential to assess the durability of microbiome and immunological changes induced by CFTR modulators—whether they are stable or fluctuate over time—and to understand their implications for disease progression and systemic health. Further research must also explore residual inflammation and the possibility of adjunct therapies aimed at immune modulation and microbiome stabilization. Equally important is the ongoing surveillance for systemic complications, which will become ever more relevant as the population ages. The interactions among immune responses, microbiota, and systemic health are complex, and unraveling these relationships will be key to developing targeted interventions that promote not just longevity but quality of life [67].

As outlined in Table 2, the future research directions emphasized the key challenges and proposed strategies crucial for advancing personalized and integrated care in cystic fibrosis.

## 5. Conclusions

In conclusion, while CFTR modulators have marked a new era in cystic fibrosis care, their full implications extend far beyond lung function. They open new research avenues focused on immune regulation, microbiome health, and systemic wellness. The future of CF management lies in adopting this broader perspective, aiming for a personalized approach that anticipates and addresses the multisystemic nature of the disease in an era of extended survival.

## Figures and Tables

**Table 1:** Key Themes in Modern Cystic Fibrosis Management—Insights from Immunology and Microbiome Research.

Aspect	Core Findings/Themes	Implications for CF Care
Impact of CFTR Modulators on Lung Inflammation	Significant reduction in inflammatory mediators, neutrophil activity, and immune cell dysregulation	Shift from symptomatic to targeted therapy, emphasizing personalized immune modulation
Microbiome Dynamics in the Era of Modulators	Increased microbial diversity, decreased dominance of pathogenic bacteria, potential for microbiota-based therapies	Promoting microbiome resilience as part of holistic CF management
Systemic Complications Arising from Long-Term Therapy	Rising risks of cancers, metabolic, hepatic, and bone disease	Necessity for integrated, multidisciplinary monitoring aligned with evolving systemic health profiles

**Table 2:** Strategic Directions for Future Systemic and Immunological Research in CF.

Theme	Key Challenges	Innovative Solutions & Research Priorities
Long-Term Safety & Systemic Risks	Unknown long-term effects on immune surveillance, cancer risk	Establish global CF registries, conduct longitudinal, systems-level studies
Residual Inflammation & Microbiome Stability	Persistent immune activation, microbiome fluctuations	Develop adjunct immunotherapies, microbiota-targeted interventions
Personalized & Integrated Care Approaches	Tailoring management to evolve systemic and pulmonary profiles	Advances in biomarkers, multi-omics, personalized medicine strategies

## Data Availability

Not applicable.

## Abbreviations

The following abbreviations are used in this manuscript:

CF	Cystic Fibrosis
CFTR	Cystic Fibrosis Transmembrane Conductance Regulator
FEV1	Forced Expiratory Volume in 1 s
IL	Interleukin
CFRD	Cystic Fibrosis-Related Diabetes
